# Population versus individual protection by pneumococcal conjugate vaccination

**DOI:** 10.1016/S0140-6736(19)30039-X

**Published:** 2019-05-25

**Authors:** Keith P Klugman, Gail L Rodgers

**Affiliations:** Bill & Melinda Gates Foundation, Seattle, WA 98119, USA

In *The Lancet*, Laura Hammitt and colleagues^1^ describe a reduction of pneumococcal disease burden in all ages by childhood immunisation with ten-valent pneumococcal conjugate vaccine (PCV) in Kenya, a low-income country (LIC), which adds to the body of evidence for this effect, which is well described to date only in high-income^2^ and middle-income^3^ countries.

Their longitudinal surveillance study established a comprehensive clinical and microbiological surveillance system within the Kilifi Health and Demographic Surveillance System (KHDSS) that encompassed a population of 284 826 in 2016, where blood and cerebrospinal fluid were obtained using a detailed protocol in individuals admitted to the sole government hospital; cross-sectional nasopharyngeal (NP) carriage surveys were conducted annually on 500 randomly selected individuals of all ages; and vaccination data were linked to children’s identification in the KHDSS at the 26 clinics administering vaccines. Using this thorough surveillance system, Hammitt and colleagues^1^ show significant reductions in vaccine-type (VT) invasive pneumococcal disease (IPD) and VT carriage in all age groups, although VT carriage persisted in 6% of children younger than 5 years of age. In Hammitt and colleagues’ study, introduction of the ten-valent pneumococcal conjugate vaccine with a catch-up campaign for children younger than 5 years and without a booster dose reduced the incidence of VT IPD in children aged up to 5 years by 92% (95% CI 78–97) and in unvaccinated children by 74% (41–89) in the 5–14-year age group and by 81% (49–93) in the 15 years and older age group. The strength of this study lies in the establishment of the surveillance and laboratory systems, and their linkage to a well functioning health and demographic surveillance system, leading to high-quality, reliable evidence of vaccine effectiveness. The major limitation to extending this study to other LICs is that although direct measurement of changes in IPD is important to assess vaccine effectiveness and the effect of serotype replacement,^1–^^3^ the need for a well established infrastructure that includes policies to obtain blood and cerebrospinal fluid samples and sophisticated microbiology laboratories, presents a formidable challenge in many LICs. NP carriage is easier to detect and evaluation of reductions in VT carriage, as also reported by Hammitt and colleagues,^1^ can provide a surrogate marker of PCV effectiveness and herd protection in LICs.

Hammitt and colleagues^1^ show the effect of the PCV10 programme on IPD and the similar effect on NP carriage, both in vaccinated and unvaccinated populations, thus adding to extensive data that herd protection is induced by prevention of transmission of PCV serotypes, which is mediated by reduction in carriage. This study also supports the evidence that reduction in VT carriage among children aged 3–5 years, who were part of the catch-up population in Kilifi, might be the best predictor of reductions in invasive pneumococcal disease among all ages.^4^ The importance of these inferences from Hammitt’s study is that they would allow cross-sectional measurement of VT carriage in children aged younger than 5 years following PCV programme implementation, which is a valuable tool, and perhaps the only widely accessible tool to assess new strategies to sustain immunisation programmes in LICs, especially those graduating from financial support from Gavi, the Vaccine Alliance.

As noted by Hammitt and colleagues,^1^ the residual VT colonisation in Kilifi is higher than that seen in high-income countries such as the USA^5^ and the UK,^6^ where booster dose-containing schedules, either three or four doses (2 + 1 or 3 + 1), lead to residual VT carriage in less than 5% of children younger than 5 years. A rapid decrease in VT carriage was seen in this age group in South Africa, a middle-income country, where only 1 year after implementation of a three-dose booster-containing schedule, VT colonisation in children aged 3 months to 2 years decreased from 40. 8% to 13.8%.^7^ By contrast, data from Mozambique 2 years post implementation of a three-dose PCV regimen administered at 6, 10, and 14 weeks of age without a booster and without catchup (3 + 0) showed a residual VT carriage prevalence of 20.7%.^8^ Even in more mature PCV programmes in Malawi^9^ and The Gambia,^10^ which use a 3 + 0 schedule, residual VT carriage remains high at 16.5% (3 years after introduction) and 11.4% (5 years following introduction). The 6% residual VT carriage described by Hammitt and colleagues in children younger than 5 years in Kilifi may be secondary to rapid induction of herd protection due to catch-up vaccination of older children aged 1–5 years, despite the lack of administration of a booster dose.

**Figure:**
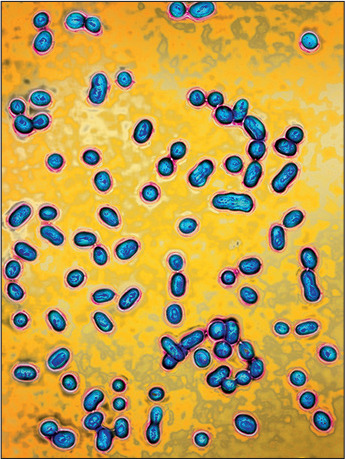
James Cavallini/Science Photo Library

Although the benefits of a catch-up PCV programme are apparent here in Kilifi, contributing to data that have led Gavi to belatedly accept catch-up as an introduction strategy, the long-term effect of this approach on residual VT transmission is unknown, and a more affordable long-term approach, such as a 2 + 1 strategy, might be needed to achieve and maintain population protection. In countries with low levels of VT disease and carriage, a further iteration of population protection might be to eliminate a further infant dose for a 1 + 1 strategy, as suggested by the UK^11^—provided that the second dose is a booster—to maintain population protection through low levels of VT carriage in children. For countries without boosters, a population-based approach might be to first change to the three-dose booster containing a 2 + 1 regimen to achieve and then maintain carriage of VT at low levels, followed by the two-dose booster containing a 1 + 1 regimen, which might provide a more sustainable and affordable option. In support of this idea, the Bill & Melinda Gates Foundation is funding studies to evaluate the immunogenicity and effect on carriage of the two-dose (1 + 1) regimen in The Gambia, India, South Africa, and Vietnam. Thus far, similar immunogenicity to that seen in the UK of this two-dose regimen has been shown in South Africa (Shabir Madhi, Medical Research Council, Respiratory and Meningeal Pathogens Research Unit, University of the Witwatersrand and Department of Science/National Research Foundation: Vaccine Preventable Diseases, Faculty of Health Science, University of the Witwatersrand, Johannesburg, South Africa, personal communication). Further data on VT disease and carriage will, we hope, elucidate the cost–benefit criteria for a two-dose future comprising more affordable, booster-containing PCV regimens for maintenance of pneumococcal VT herd protection in LICs.
